# Quantifying the retention of emotions across story retellings

**DOI:** 10.1038/s41598-023-29178-8

**Published:** 2023-02-11

**Authors:** Tianyou He, Fritz Breithaupt, Sandra Kübler, Thomas T. Hills

**Affiliations:** 1 Department of Psychology, University of Warwick, Gibbet Hill Road, Coventry, CV47AL UK; 2 Department of Germanic Studies, Indiana University, Bloomington, USA; 3 Cognitive Science Program, Indiana University, Bloomington, USA; 4 Department of Psychology, University of Warwick, Gibbet Hill Road, Coventry, UK

**Keywords:** Computer science, Mathematics and computing, Psychology, Human behaviour

## Abstract

Story retelling is a fundamental medium for the transmission of information between individuals and among social groups. Besides conveying factual information, stories also contain affective information. Though natural language processing techniques have advanced considerably in recent years, the extent to which machines can be trained to identify and track emotions across retellings is unknown. This study leverages the powerful RoBERTa model, based on a transformer architecture, to derive emotion-rich story embeddings from a unique dataset of 25,728 story retellings. The initial stories were centered around five emotional events (joy, sadness, embarrassment, risk, and disgust—though the stories did not contain these emotion words) and three intensities (high, medium, and low). Our results indicate (1) that RoBERTa can identify emotions in stories it was not trained on, (2) that the five emotions and their intensities are preserved when they are transmitted in the form of retellings, (3) that the emotions in stories are increasingly well-preserved as they experience additional retellings, and (4) that among the five emotions, risk and disgust are least well-preserved, compared with joy, sadness, and embarrassment. This work is a first step toward quantifying situation-driven emotions with machines.

## Introduction

Ever since the advent of human language, story retelling has been a crucial mechanism in the conveying of information and evolution of culture^1^. Lessons learnt from past experiences were transmitted down the generations, such that individuals encountering similar situations stood a greater chance of survival. As humans began organising into sustained social groups, story retelling manifested into the role of propagating cultural norms, ideas, and entertainment, all of which engender cooperation and cohesion—integral to an evolving civilisation^2,3^. A core aspect of this communication by means of storytelling are emotions, as they indicate how one could react to the given information, including approach or avoidance behaviour^4^. It has been shown that emotions expressed on the internet influence subsequent communication^5^. There are thus good reasons not just to consider the adaptive features of storytelling in general^6,7^, but also emotions in narratives specifically^8^. People are able to co-experience past or imaginary situations by means of stories; to study story retelling is thus to better understand the fundamental substrate of communication within human civilisation^9^.

There is a wide range of evidence that emotions play central roles for stories and storytelling. Emotions have been shown to influence people in the creation of stories, as Rao et al.^10^ demonstrated—that by priming participants with different types of emotions, the stories produced by these individuals become richer. Stories show specific emotional arcs that can be measured by word-based sentiment detection^11,12^. Nabi and Green^13^ suggest that emotional flow–how emotions change within a narrative–intensifies immersion within the narrative world (i.e., transportation). Emotions play a key role in the effectiveness of narratives for the audience^14,15^. Emotions also stimulate the sharing of narratives^16,17^. In general, social information prevails in story retelling and is often paired with emotions^3,18^. Regarding story retellings, Breithaupt et al.^19^ suggested that there exists an emotional dimension guiding storytellers and providing stability to their retold stories. They found evidence for the preservation of surprise even when the factual information is altered from the original story during serial reproduction. Breithaupt et al.^19^ proposed that the communication of emotions is an implicit goal of story retelling, with the aim to create a particular emotional response in the audience.

Not all models of narratives consider emotions as relevant elements. Many models focus on the factual information of who did what to whom, why, when, where and how^20–23^ or the identification of event boundaries^24^. Following Breithaupt et al.^19^, we propose that emotions are central to narrative communication: a key component of narrative coherence is maintained in the emotion it communicates to its audience. We follow Frijda et al.^25^ in thinking of a narrative as communicating an “emotion episode,” and critically that it is the elicitation of a specific emotional response in the recipient that is a key feature of narratives which story tellers aim to preserve in their retellings.

Critically, the emotions engendered by narrative “emotion episodes” exist independently from the specific events or emotion words used in stories. In a sentence like “With tears in his eyes, Joe is holding his crying new-born baby for the first time”, many readers of this episode will recognize this situation as happy despite the tears of Joe and his baby. Similarly, when a story character blindly steps over an open manhole, the audience is intended to feel the inherent threat even when the character does not. Thus, the emotional signature of a narrative may be independent of what the characters in the story feel.

How can we evaluate the emotional continuity of stories and their retellings? With recent advances in natural language processing (NLP), there is now a greater opportunity to study to what extent story retellings preserve emotional content. The advance most relevant to this question is the representation of meaning via automatically acquired vector space representation, generally called word embeddings. Popular versions of these models include the skip-gram and continuous bag-of-words (CBOW) models by Mikolov et al.^26,27^, which Le and Mikolov^28^ then extended to the document level. These embeddings represent an abstraction over single words so that related words are automatically grouped together without any human guidance. Hollis and Westbury^29^ found evidence that these embeddings mapped words along lexical and semantic concepts that were standard in psycholinguistic research, without explicit supervision in these concepts. However, skip-gram and CBOW models are limited in the amount of information they can represent. One limitation of these models is that they cannot capture long range dependencies between words in longer sentences. Additionally, they lack the ability to account for polysemy: When words possess different senses in different contexts, they are still represented as single embeddings.

Numerous proposed models followed to address these limitations, with the most notable being the transformer models, introduced by Vaswani et al.^30^ Their seminal work formed the basis for a range of state-of-the-art NLP models, with Bidirectional Encoder Representations from Transformers (BERT) by Devlin et al.^31^ being one of the most influential. BERT can represent words in context, meaning that it can distinguish between different senses of a word. This model had been shown to produce state-of-the-art performance in a multitude of NLP tasks. In the task of emotion detection, Huang et al.^32^ found that tuning BERT to the dialogues in Facebook Messenger chat and to the Friends television sitcom series allows the model to recognise the presence of the emotions of joy, sadness, and anger with excellent performance. In a recent review by Acheampong et al.^33^, which tracks the progress of state-of-the-art approaches in text-based emotion detection, the approaches by Huang et. al. were among those highlighted.

However, BERT is still significantly undertrained as a multi-purpose model for NLP. Consequently, Liu et al.^28^ introduced the Robustly Optimized BERT Pretraining Approach (RoBERTa). They demonstrated that by slightly tweaking the BERT architecture and exposing it to more text data, RoBERTa significantly outperforms the BERT model. This makes RoBERTa an extremely powerful NLP tool for research involving text data.

The present study uses RoBERTa to evaluate to what extent emotions are preserved across a unique corpus of multi-generation story retellings (*n* = 25,728) from Breithaupt et al.^34^. This corpus was collected from the iterated retelling of seed stories (*n* = 100), distributed across five factors (embarrassment, joy, sadness, disgust, and risk)—four emotional factors and one situational factor (risk). “Risk” or riskiness was included in the original study^34^, and we included it in our analysis, as well. According to the risk-as-feeling hypothesis of Loewenstein et al.^20^, risk is a response emotion in that people have emotional responses to risky situations, which they in turn use to evaluate relative risks. For example, people may feel fear, worry, or anxiety when a character walks unknowingly through a minefield, and the degree to which they have those emotions is directly correlated with the degree to which they perceive the situation as risky. Following this risk-as-feelings framework, we included risk in our study to examine the preservation of risk and to what extent RoBERTa can detect risk, a finding with potentially wide applications (e.g., in threatening online communication and legal contracts designed to ameliorate risks).

These stories are also provided with human ratings for the intensity of each of these factors. The emotions and also risk are not named in the stories, but were designed with the intention to create varying thematic emotion episodes in the reader^25^. In the case of sadness, this might be the loss experienced by a character; in the case of happiness, this includes ending a negative situation, progress toward a goal, or expanding social relations; in the case of embarrassment, characters experience a social faux pas; in the case of disgust, characters face a repulsing situation, such as a messy roommate or indigestible food; in the case of risk, this includes dangers of loss via financial investments or the potential of physical harm when performing actions.

Using this corpus, the present study aims to leverage the power of RoBERTa to encode emotion-rich story-level embeddings. The purpose is both to evaluate the ability of artificial intelligence to predict emotional responses to stories (trained on data from human rates) and to evaluate the extent to which story tellers preserve emotions over repeated retellings. The current study thus follows the study by Breithaupt et al.^34^ with different analytical tools and different emotions. However, it differs substantially from that study by evaluating the capacity for RoBERTa to encode emotions that are not explicitly named, but appear in complex contexts. In addition, this study also includes substantial data and collected stories excluded by Breithaupt et al.^34^. The analysis will focus on elucidating the evolution of emotions in stories as they are retold. The goal is to address two interlinked research questions:

### RQ1: Can RoBERTa encode emotions into embeddings sufficiently to identify the emotional content of novel stories?

As noted above, Huang et al.^32^ were able to demonstrate that a fine-tuned BERT model can discern emotions of happiness, sadness, and anger with excellent performance; thus the superseding RoBERTa model should have little challenge dissociating the stories of joy and sadness from our corpus of retellings. We extend the inventory of emotions and add three more emotions present in this corpus, namely embarrassment, risk, and disgust. Considering that RoBERTa was pre-trained on over 160 GB of textual data from book, Wikipedia, news, web, and story corpora, the likelihood of RoBERTa having encountered these emotions is high. It is therefore expected to perform reasonably well at this task. An additional challenge for RoBERTa is introduced since we expect the model to identify not only the emotion, but also the emotion intensity from a story. However, the main difference to prior investigations comes from the type of stories in our corpus: In the stories, the emotional categories are not named explicitly in the texts, but are implicit in the story situations, as in many real-life communications. For example, being offered a job tends to be an event causing happiness; spilling wine on the shirt of one’s boss is embarrassing. If RoBERTa can learn to reliably recognize emotions and their intensity from the description of events, then the resulting embeddings will be sufficient to detect emotions in novel stories. Thus, our first research question aims to assess whether this is the case. Only if this is true, the embeddings can be used to address the second research question of this study.

### RQ2: Do individuals on average preserve emotional qualities of a story during the process of retelling?

When retelling a story, do people have the tendency to preserve the emotional “centre of gravity”? This is in reference to the concept of stability proposed by Breithaupt et al.^19^ who found that the emotion of surprise was preserved in retellings while other, factual information deteriorated. This finding could be interpreted in line with the concept of emotion episode by Frijda et al.^25^. Our hypothesis is that there exists a fundamental emotional “centre of gravity” that people agglomerate towards in their retellings, such that the central emotion in a story is preserved over retellings. Because RoBERTa allows us to quantify this preservation, we can also investigate the extent to which the five emotions represented in the corpus differ from one another.

## Data and methods

### Corpus

This study leverages the unique corpus of story retellings from Breithaupt et al.^34^. The data set offers a large set of stories that contain variations of some basic seed stories, controlled to model the specific emotion conveyed as well as the emotional intensity. Retellings of basic stories lead to variations that afford us the opportunity to analyse the effects of different variations.

The dataset was constructed from a set of seed stories, three stories per emotion. Each story had a lead character (e.g., Avery) and 5–8 variants differing in the emotion intensity of the ending. These variations of the seed stories were retold by 11,801 participants who were instructed to read multiple stories and retell them in their own words. The corpus was initialised by seed stories of an average length of 182.7 words (Generation 0) that were retold by different participants in chains to result in 3 generations. As in the telephone game, each reteller only saw the version directly before (for this method of serial reproduction, see Bartlett^35^; Kashima^36^; Mesoudi and Whiten^37^).

The seed stories were generated by the researchers across five types of emotions; namely joy, sadness, disgust, embarrassment, and risk. Each story ends in a situation that transmits one of these specific emotions, such as someone crossing an icy bridge (risk), and different story endings were created with varied intensities of the emotion from low to high. Raters confirmed that these variations resulted in a spectrum from low to high ratings for a given emotion. For example, one basic story sets the scene for a lonely student who had her computer stolen in college. A low happiness variation of that story results in an improvement of the weather (emotion rating for joy of 2.64 on a scale from 0 to 7), while a more joyful variation ends with her falling in love (emotion rating of 5.26 on a scale from 0 to 7). This process transformed 15 basic story sets, with three for each emotion, into a total of 97 different stories to be used as seeds.

For the retellings, participants within each generation were randomly allocated three stories of distinct emotions originating from distinct seed stories. They were then instructed to retell their three stories that would take the original stories’ place for the next generation of retellers. At no point were participants primed or instructed to pay specific attention to a story’s emotion, rather they were simply asked to retell each story in their own words. The seed stories did not contain the category-specific emotion words, but culminated in situations connected with specific emotions, with different levels of emotion communicated by different alternate endings. For example, below we show one version of a story around risk (one of the alternate endings specified in square brackets), which received the highest average human rating for ‘risk’:

Taylor has recently been looking into the stock market. His closest friend, Frank, nearly doubled his salary by investing all his savings in different stocks. What's more, Taylor's aunt, Melinda, has also earned a lot of money by investing in a small company that became very successful. Financially, Taylor is in the green. He has some money in a savings account at his bank, has a steady job, and has recently received an inheritance from his late grandmother, who passed away last spring. On his birthday, he hit the casinos and spent hours at the blackjack table before coming out with some winnings. Listening to stories from his friends and family, Taylor feels excited about putting his money to work for him, and thinks that he should begin investing. He begins looking into different companies in which he can invest during his free time and eventually finds a green energy startup company that he thinks looks rather promising. He invests [all his savings, and takes out a large loan with the bank to invest even more.]

A total of 25,728 retellings were collected across all three generations based on stories such as the one provided above. Our analysis offered in this paper includes duplicates of retellings initiated by the same previous story that were excluded in the analysis by Breithaupt et al.^34^ since they focussed on retellings with complete single chains and thus only analysed 18,738 of the collected retellings.

To retrieve emotion intensity scores, 5234 participants were each given 15 stories at random (but from within the same generation to make length comparable) for evaluation. They rated each story on a scale from 0 to 7, representing low to high levels of the associated emotion. Participants who read stories about joy, for example, were asked “how happy is this situation?”, then given a slider to rate the emotion intensity. While a situation “is” not happy/sad/disgusting/embarrassing, we reason that raters equated the question with “how happy is this situation *for a typical person in that situation*?” This assumption seems likely since the stories focussed on joy, sadness, disgust, and embarrassment suggest high convergence between character, situation, and reader position. Raters thus likely applied an overall emotion rating to the story situation with character emotions, situations, and their own feelings about this situation being similar. In the example with the lonely student, the raters likely would perceive the new situation of the student as happy, rate her as happy, and also themselves feel happy to a similar degree. The case is different for risk. In the case of risk, the question “how risky is the situation?” is grammatically correct since situations carry varying degrees of risk, but here the risk-feelings of the characters and the situation as a typical person would perceive it, diverge. This is clear in the example above where Taylor is “excited” to make an investment, while readers may likely perceive it as risky. Other measures such as presence and coherence were also recorded, but for the purpose of this study, focus was directed onto the measure for emotion intensity ratings. For all other information about the methods, see Breithaupt et al.^34^ and complete details at https://osf.io/nbuxg/.

A preliminary text analysis found that the diversity of vocabulary increases at each generation of retellings. Excluding stop words, a total of 878 dictionary words were used to construct the seed stories. The size of this vocabulary grew to 3581 unique dictionary words in generation one, which grew further to 5548 unique dictionary words in the second generation. There was a slight decrease to 5233 dictionary words in the final generation of retellings. With regards to word count, seed stories had an average of 182.7 words, while generation one retellings had an average of 87.2 words. The average in generation two was 65.6 words, and 50.2 words among the retellings in generation three.

### Robustly optimized BERT pretraining approach (RoBERTa)

To quantify the extent to which emotion is preserved across retellings for the five different emotions and three intensities, we used the RoBERTa architecture^38^, which is an extension of BERT^30^. BERT is a neural network architecture that builds on the concept of a transformer architecture to create a deep model that is highly efficient at encoding massive volumes of textual data^2^. More specifically, BERT consists of the encoder part of a transformer. It reads a text in its entirety instead of incrementally, which makes it bidirectional since it has access to the context to the left and right of a word that it is processing. This architecture allows BERT to learn embeddings that represent word senses rather than words. In order to make the embeddings more robust, BERT is trained on two tasks, a language model in which the model needs to predict masked words, and a task where the model needs to predict whether two sentences are adjacent in a text. The first task forces the model to focus on the word level while the second task gives it a view of larger contexts. RoBERTa, in contrast, only uses the first task, but modifies it so that the masked tokens change dynamically during the training process.

BERT was pretrained on 16 GB of text data from a book corpus (800 million words), and the English Wikipedia corpus (2500 million words). RoBERTa is a retrained model of BERT with a considerably larger textual base: it uses an additional 145 GB of text, ranging from news, web, and story corpora. This can be interpreted as feeding vast amounts of the English language to RoBERTa, and having it represent their intrinsic structures into a multi-purpose NLP model that can be fine-tuned for task-specific purposes.

In general, neural architectures can be retrained on different tasks. In this case, the output layer, which is specific to a task, is replaced by a new layer representing the new task. If the previously learned representations are useful for the new task, the architecture can then be retrained with a small training set of the new task. For the purpose of this study, RoBERTa needed to be retrained to recognize the 5 emotions unique to the retellings corpus in order to encode them into embeddings appropriate for this study. This was achieved by tuning RoBERTa towards the task of classifying both the emotion and its intensity for a given retelling.

Prior to the fine-tuning phase, the emotion ratings were converted from the 0–7 scale into low, moderate, and high factor levels, thus converting the problem from a regression problem to classification. Performing regression in BERT style models has not been investigated in depth, thus we leave this problem for future work. Stories rated lower than 2.33 (1/3 of the maximal value) were assigned to the low factor level while stories with ratings higher than 4.67 (2/3 of the maximal value) were assigned to the high factor level. Stories with ratings that fell between that range were assigned to the moderate factor level. These factor levels were then combined with the associated emotion class of a given story to create 15 one-hot encoded classes (e.g. Low Sadness, Moderate Risk, High Joy, etc.). This process was repeated for every generation of retellings. The reason behind combining emotion with intensity was to increase the difficulty of the task, such that (1) RoBERTa was encouraged to dissociate emotion intensities, and (2) we avoided having the model predict emotion by simply memorising character names in a given story. Furthermore in the process of retelling, the same story in a chain of retelling may cross over between different factor levels; for example, a story that was rated as moderate for the first generation may have resulted in some retellings that would deviate either into the high or low factor levels (note that each retelling was individually rated by human raters for intensity). This deviation in intensity suggests that the specific words associated with specific story endings (which correspond to the different intensities in the original stories) would not provide reliable information about the factor level. Consequently, these challenges forced the model to produce more robust embeddings.

The pretrained 50 layered RoBERTa architecture^39^ was used, which was stacked with a dropout layer, with a 30% dropout probability for regularisation. A final output layer with 15 neurons was then added to match the dimensions of the classification task. Binary cross entropy was defined as the loss function, with training and testing batch sizes set to 32. The Adam algorithm^40^ was used as the optimiser, and the model was trained over 5 epochs, at a learning rate of 0.00001 on all 8576 retellings in generation one. Generation one retellings were used as the training set as it was the point of first contact from participants following the researcher-generated seed stories. This increased the likelihood of better gradation among emotion rating scores, and it fostered greater diversity in terms of textual content. Performance of the tuned model was validated on generation two retellings, before having its performance evaluated across all generations, using accuracy, macro-averaged F1 (macro-F1), and micro-averaged F1 (micro-F1) scores as evaluation metrics.

### Determining similarity between story representations

In order to answer our research questions, we need to first gain access to the representation of a story from RoBERTa, and then to determine the similarity between a story and the retellings.

To access the representation of a story, embeddings were retrieved from RoBERTa’s last hidden layer, which has a total of 768 nodes, i.e., we retrieved a vector of 768 dimensions. This means that we ignored the classifications produced by RoBERTa, which are generally the intended use of such a model. Instead, we accessed the internal representation in the last hidden layer.

In order to determine the similarity of story variants, we used two methods, principal components analysis and cosine similarity. Principal components analysis is a standard methodology to reduce the high number of dimensions to a smaller number of highly relevant dimensions. It is used, for example, in latent semantic indexing^41^ or generalising skip-grams^42^, and can be used to improve human interpretability. Cosine similarity, in contrast, is an established method for determining the similarity between high-dimensional vectors which is often used in information retrieval and text mining^43^. Here, the two metrics allow two complementary views on story similarity.

Principal component (PC) analysis was performed on the extracted embeddings to ascertain whether the retellings organised themselves along dimensions of emotion. The original 768 dimensions of all embeddings were projected onto the number of dimensions capturing 99% of explained variation. The PC scores for each principal component were then plotted against their corresponding emotion rating, and colour coded by their associated emotion.

Fundamentally, cosine similarity (CS) considers the angle between two high-dimensional vectors, and determines a similarity score ranging from − 1 to 1. A score of 1 would mean that the two vectors point in the same direction within a vector space and are thus very similar, while a CS of − 1 indicates that both vectors are pointing in the exact opposite direction and are thus very dissimilar. Another important property of CS is its independence from document length^44^, which is especially relevant to this study, given that the average length of stories decreased at each retelling generation. Thus, using CS as a similarity metric is unlikely to introduce bias from the retelling word counts. Once a high correspondence of RoBERTa embeddings to emotion qualities was ascertained via the first research question, pairwise CS scores on the 768-dimensional embeddings were collected from both within generation and between generation treatments, to answer the second research question.

For the within-generation treatment, because each of the 97 original seed stories had different vocabulary, pairwise CS scores were only collected for each paired combination of stories that shared the same original seed. For example, every retelling within a generation that originated from one story version about the character ‘Avery’, were scored with each other—ignoring redundant comparisons and self-similarities. There was an average of 88 stories sharing the same original story seed (meaning retellings of the seed story in generation one or G1), resulting in an average of 3828 total pairwise combinations per seed, excluding self comparisons. Formally, for N stories that share a seed, the N × N cosine similarity matrix representing all possible pairwise combinations of N was computed, where N(N − 1)/2 elements were collected from the upper triangular portion, less the diagonal of that matrix.

For the between generation treatment, pairwise CS scores were computed across 5 generation contrasts (G0vsG1, G0vsG2, G0vsG3, G1vsG2, G2vsG3), along the serial path of retellings. These generation contrasts were collated into two subsets of sequential contrasts. SET 1 [G0vsG1, G0vsG2, G0vsG3] looks at the scores between the seed story and its retold outcomes in each of the following generations. SET 2 [G0vsG1, G1vsG2, G2vsG3] looks at the transitional scores between each generation and its successor. If CS scores are maintained across sequential contrasts in SET 1 and SET 2, then that supports the hypothesis that the emotions remained stable through the retelling process.

In general, we report accuracy, micro- and macro-averaged F1 scores. Accuracy in itself can be misleading since it emphasises the performance per retelling, thus potentially hiding if certain categories are more difficult than others. F1 is the harmonic mean of precision and recall, a standard measure originally introduced to evaluate information retrieval^45^. It ranges between 0 and 1. The micro-averaged F1 is weighted by category frequency, the macro-averaged F1 averages over categories rather than stories. For this evaluation, RoBERTa is treated as one rater and the human raters as the other. To evaluate how CS scores changed in response to emotion types and retellings, Type-3 ANOVA and follow-up tests were conducted with the CS scores as the dependent variable, with emotions as the between factor, and the generation contrasts as the within factor.

### Ethics statement

Ethical approval for this work was granted by the Indiana University IRB review board. IRB-IUB, IRB00000222 (#IRB-IUB, IRB00000222). All methods were performed in accordance with the relevant guidelines and regulations.

## Results

### RQ1: Story embeddings from a fine-tuned RoBERTa model show high degree of correspondence with human ratings of emotions

RoBERTa’s task was to classify a given retelling into one of fifteen classes, represented by 3 levels of emotional intensities for 5 different emotions. A random classifier in this case would only achieve an average accuracy of 6.67% for all generations. In contrast, for the seed generation, our tuned RoBERTa model was able to achieve an accuracy of 78.28%, a macro-F1 score of 70.76% and a micro-F1 score of 78.28%. The scores for second generation retellings were 64.34%, 62.96%, and 64.34% respectively. For generation three retellings, the accuracy was 62.89%, with 59.4% for its macro-F1 score, and 62.89% for its micro-F1 score. For completeness, the model’s performance on training data reached an accuracy of 97.14%, 96.61% for macro-F1 score, and 97.14% for micro-F1 score. These results demonstrate that RoBERTa appropriately encoded the affective emotions surrounding the retellings.

**Figure 1:**
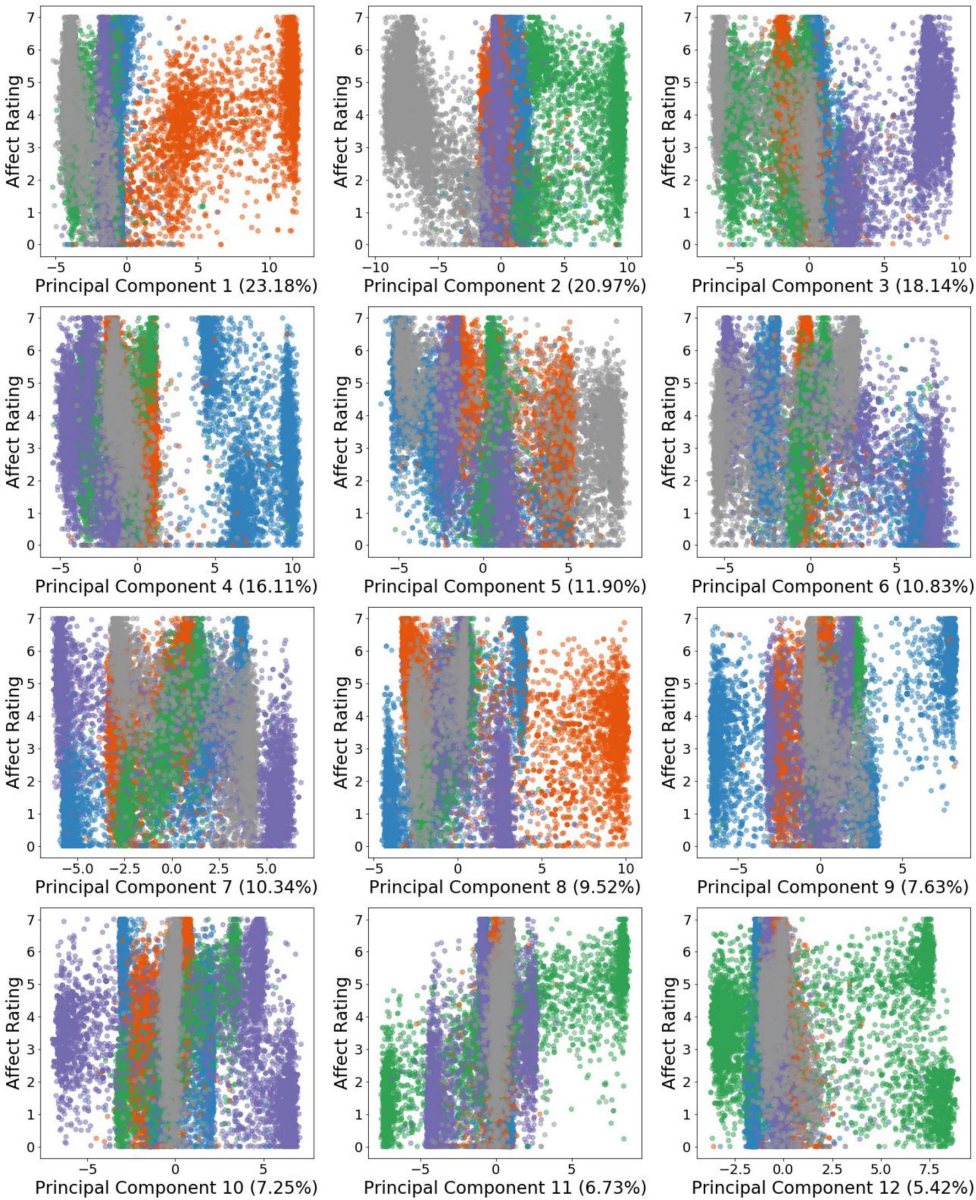
Scatterplots for each of the first twelve principal components (PC) ranked by explained variance (in brackets), from all fine-tuned RoBERTa embeddings. Each subplot plots the projected PC scores on the x-axis against their corresponding emotion ratings on the y-axis. Every observation is colour-coded by emotion, with orange representing embarrassment stories, grey for sadness stories, green for joy, purple for risk, and blue for disgust stories.

To further demonstrate this encoding of emotion, Fig. 1 visually depicts the relationship between emotion ratings (intensity) and emotion (colour-coded) for the first 12 PCs. These 12 PCs were sufficient to explain 95% of the variance, while 27 components captured 99% of the explained variation (Figure S1 providing the remaining 27 PCs can be found in the Supplementary Material). Figure 1 shows that the first four PCs visually dissociate the five emotions, separating sadness, joy, risk, disgust, and embarrassment on alternate sides of the zero PC score. These four PCs amount to a combined total of 78.4% in explained variance.

More specifically, we can see that PC1 (the top PC that explains 23.18% of variance) separates all embarrassing stories and maps them to the right, away from all other stories on the left. Furthermore, a positive relationship was shown between the PC1 scores and the emotion rating. As these scores increase from 0 to 12.5, they become associated with stories evaluated by humans to have higher intensities of embarrassment. A different pattern emerged in PC2 such that all sad stories are presented to the left, with all joyful stories to the right. Large negative PC2 scores trend downwards to represent declining levels of sadness, while large positive scores trend in the opposite direction, aligning with increasing levels of joy. Stories from all other emotions accumulate tightly around the zero PC score. PC3 arranges all risk stories to the right, and also showcases a positive relationship between its scores and their emotion ratings such that stories evaluated with increasing intensities for risk are encountered as PC3 scores grow. PC4 distinctly separates stories of disgust, but fails to showcase a clear relationship between its scores and the human rated intensities of that emotion.

An interesting pattern also emerged from PC11, where the intensity of joy displays a clear positive relationship with PC11 scores as they increase from the far left to the far right. An intermediate relationship for the risk emotion is also apparent. It is up to speculation whether this shows that PC11 could encode an emotion overlap between joy and risk. For example, stories of risk that resulted in reward could engender emotions of great joy, while risk resulting in loss commanded less joy (Breithaupt et al.^34^ speculate that the risk stories in the corpus could have been evaluated as thrilling and thus positive). If this was truly modelled by PC11, then we could infer that RoBERTa is powerful enough to amalgamate the semantics of different emotions, without being explicitly informed about their relatedness. Considering our model’s emotion detection performance, plus witnessing the clear and dominant semantic organisation among its fine-tuned embeddings, a strong case can be made for their utility to study the core research question in this study.

### RQ2: Storytellers preserve different emotions to different degrees

We first explore the extent to which individuals altered emotions in stories, by comparing their retellings to retellings of the same story by others under the within-generation treatment. That is, for each generation, we determine by how much a given retelling differs from another. We naturally anticipate dissimilarity between retellings as individuals heterogeneously exaggerate or reduce emotional elements of a story. However, we expect that on an aggregate level, emotive qualities of retellings within each generation do not deviate drastically from one another, such that emotions are preserved. Alternatively and contrary to our expectation, there may be specific patterns of decay or amplification.

While CS scores are often used to compare embeddings, they are most meaningful in a relative sense, such that researchers observe that CS scores are larger or smaller than some comparable benchmark. For language samples, there is rarely a single perfect comparison group. However, in the present case it is useful to provide a comparison of out-of-category emotion embeddings for Generation 0. This compares the embeddings for a story (e.g., in the joy category) with all stories from other categories. This offers a useful benchmark for how large one might expect the CS differences to be if the stories were about different topics and emotions and yet still written in roughly the same format for roughly the same audiences. The mean (and SD) for these CS values are as follows for each emotion in comparison with all G0 stories from other categories: Disgust, 0.10 (0.04); Embarrassment, 0.15 (0.05); Joy, 0.12 (0.06); Risk, 0.12 (0.04); Sadness, 0.14 (0.05). If story retellings largely preserve similarity, they should be much closer to values of 1 than they are to these out-of-category comparisons.

**Figure 2:**
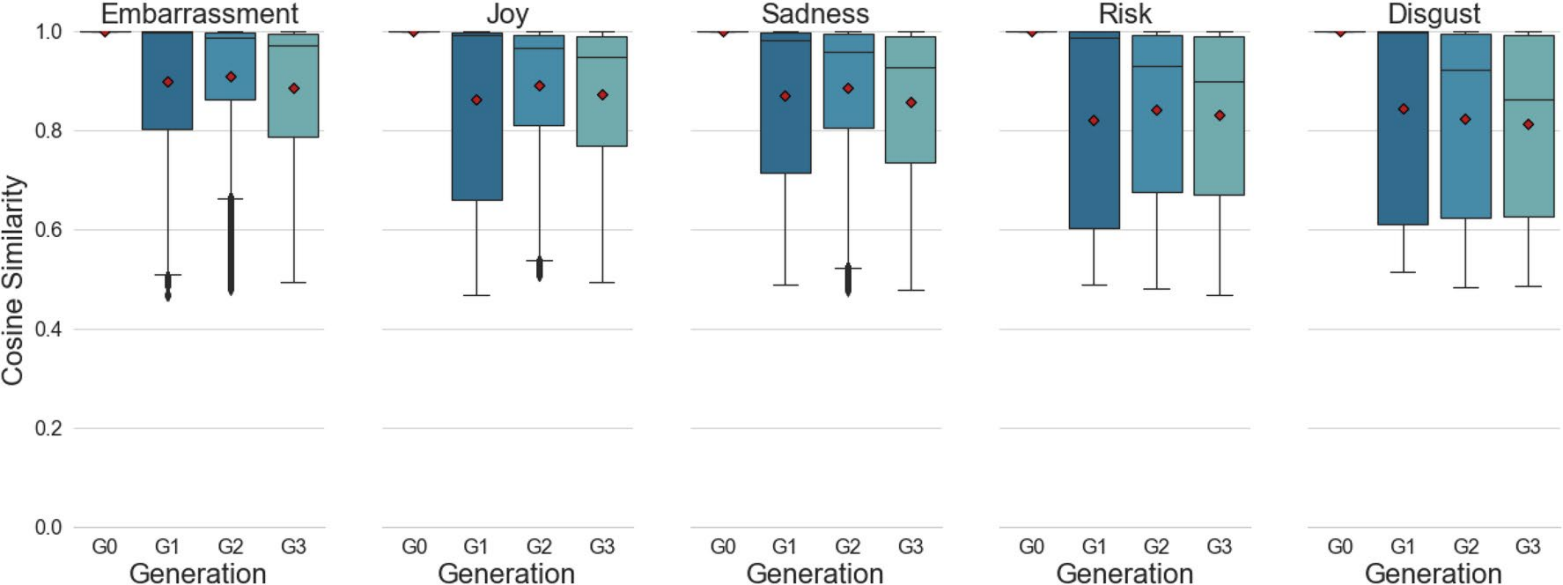
Boxplots of cosine similarity (CS) scores between stories that shared the same story seeds—within each generation. Individual means are represented by red diamonds, and outliers are represented by a black point.

The results of the within-generation comparison are shown in Fig. 2. As all seed stories are identical in Generation 0 (G0), CS scores have a value of 1. First retellings show a large variation in scores across the five emotions. The distribution is skewed downwards, with the median scores indicating a high degree of similarity (> 0.9 for embarrassment, joy, and sadness and > 0.3875 for risk and disgust). That high degree of similarity is then maintained throughout the remaining generations two and three. These results confirm our expectations that although retellings differ among each other as individuals heterogeneously alter their stories, they still maintain high degrees of similarity such that the core emotion is preserved within each generation.

**Figure 3:**
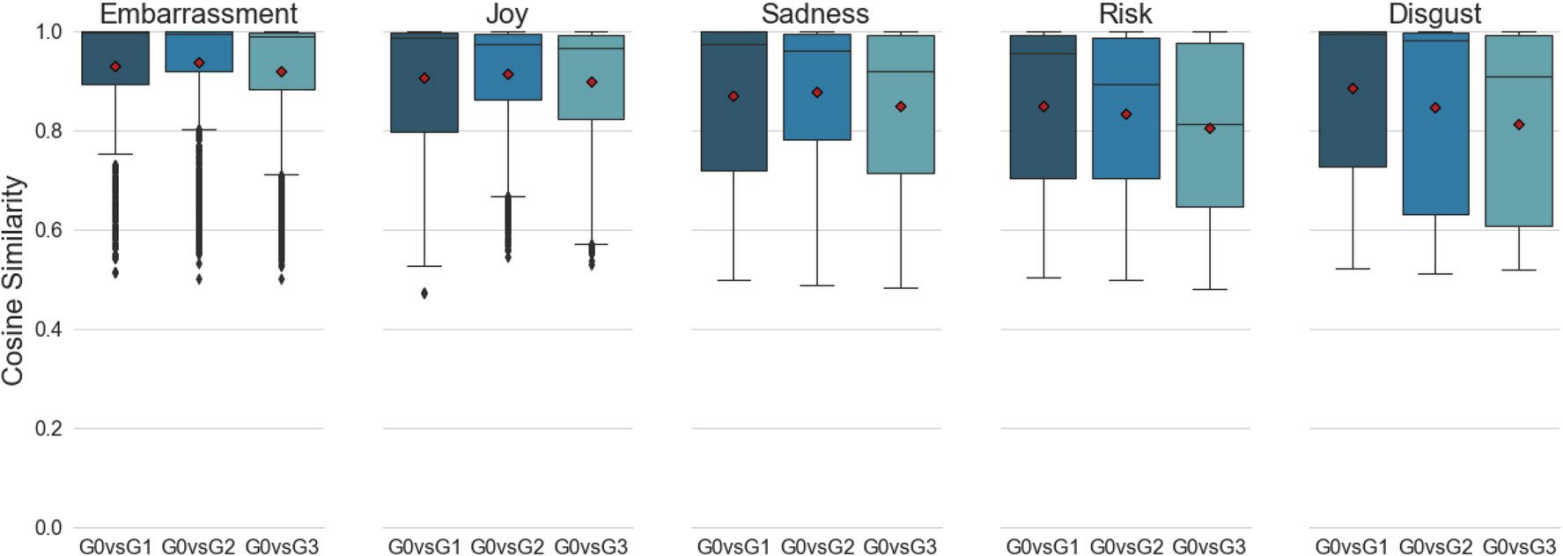
Boxplots of SET1 cosine similarity (CS) scores comparing seed stories to their retold outcomes, across different generations along the serial path of retelling. Individual means are represented by the red diamonds, and outliers are represented by a black point.

**Figure 4:**
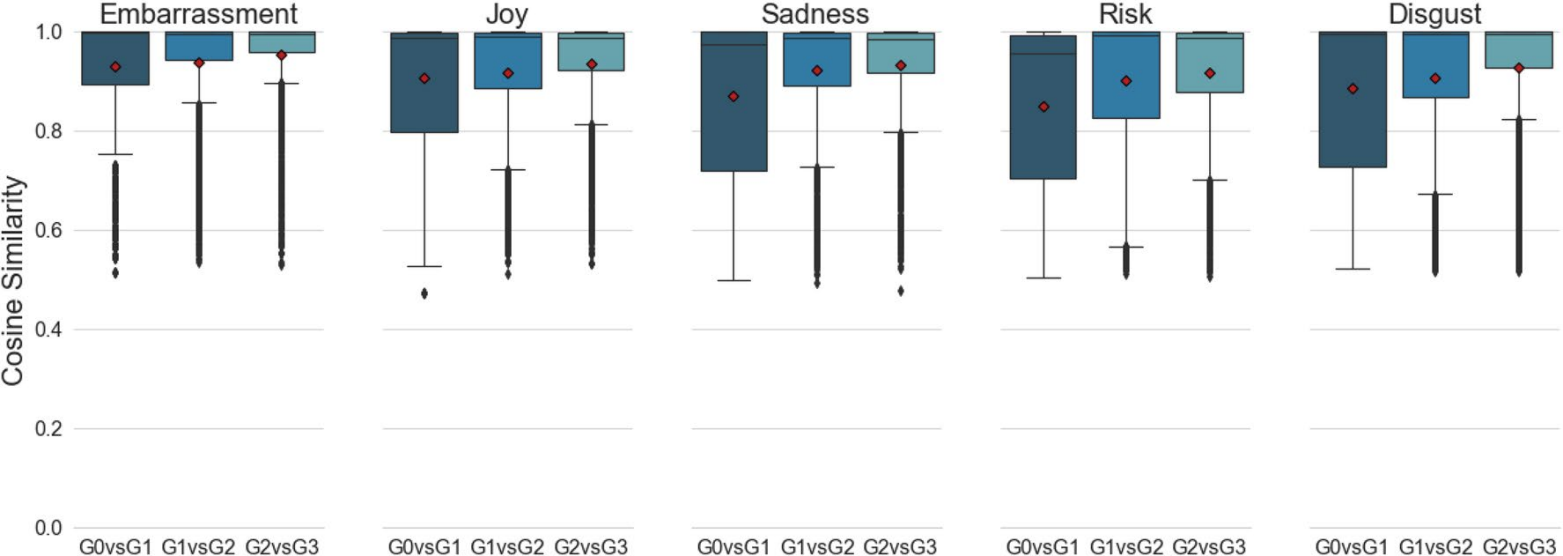
Boxplots of SET2 cosine similarity (CS) scores comparing stories between consecutive generations along the serial path of retelling. Individual means are represented by the red diamonds, and outliers are represented by a black point.

The core research question of this study is to ascertain whether emotions were well maintained throughout retellings. To answer this, we studied the differences between the CS scores under two sets (SET 1 and SET 2) of sequential generation contrasts: comparing each generation with Generation 0 [G0vsG1, G0vsG2, G0vsG3], and comparing each generation consecutively [G0vsG1, G1vsG2, G2vsG3]. Boxplots for the SET1 CS scores are visualised in Fig. 3, and SET2 scores in Fig. 4.

Both Figs. 3 and 4 show that the majority of CS scores accumulate within the high similarity ranges, with the greatest accumulation happening among embarrassment stories. The average retelling in every generation did not fall below a 0.8 CS from its seed (and are thus considerably closer to 1 than to the out-of-category values of − 0.10 and lower). Looking at mean transitional scores in Fig. 4, it can be seen that retold stories maintained high similarities to their immediate predecessors, with increasing similarity for each consecutive retelling.

An omnibus test was conducted for each set of sequential generation contrasts, using Type-3 ANOVA under sum-to-zero contrasts. For each test, emotions were set as the between factor with 5 levels, and the generation contrasts were set as the within factor with 3 levels. The test for SET1 showed significant main effects for both the emotion factor, *F*(4, 25,713) = 377.38, *p* < 0.001, and the generation contrast factor, *F*(2, 25,713) = 104.15, *p* < 0.001. Congruently, the test also showed a significant emotion by generation contrast interaction *F*(8, 25,713) = 16.34, *p* < 0.001. Similar ANOVA results were found for SET2, showing a significant main effect for the emotion factor, *F*(4, 25,713) = 102.05, *p* < 0.001, a significant main effect of the generation contrast factor, *F*(2, 25,713) = 251.78, *p* < 0.001, and a significant interaction effect between the two factors, *F*(8, 25,713) = 14.01, *p* < 0.001. These results suggest that the affective evolution of retellings vary from emotion to emotion.

15 follow up tests were conducted for each set of sequential generation contrasts to study the differences between their CS values, resulting in a total of 30 follow up tests where for each emotion in each set of sequential contrasts, the differences between each generation contrasts were tested. All *p* values were adjusted using the Bonferroni–Holm correction to control for multiple testing. The follow up test results for SET1 are summarised in Table 1, and results for SET2 are summarised in Table 2.

**Table 1:** Summary of follow-up tests statistics for cosine similarity (CS) mean differences between emotions and within generation contrasts from SET1.

Emotion	Contrasts	Estimate	Degrees of freedom	t ratio	*p* value
SET1: G0vsG1, G0vsG2, G0vsG3					
Disgust	G0vsG1–G0vsG2	0.04	25,713	7.61	< 0.001
	G0vsG1–G0vsG3	0.07	25,713	14.28	< 0.001
	G0vsG2–G0vsG3	0.03	25,713	6.68	< 0.001
Embarrassment	G0vsG1–G0vsG2	− 0.01	25,713	− 1.59	0.15
	G0vsG1–G0vsG3	0.01	25,713	1.77	0.15
	G0vsG2–G0vsG3	0.02	25,713	3.36	< 0.01
Joy	G0vsG1–G0vsG2	− 0.01	25,713	− 1.34	0.25
	G0vsG1–G0vsG3	0.01	25,713	1.53	0.25
	G0vsG2–G0vsG3	0.01	25,713	2.87	0.01
Risk	G0vsG1–G0vsG2	0.02	25,713	2.99	< 0.01
	G0vsG1–G0vsG3	0.04	25,713	8.84	< 0.001
	G0vsG2–G0vsG3	0.03	25,713	5.85	< 0.001
Sadness	G0vsG1–G0vsG2	− 0.01	25,713	− 1.63	0.10
	G0vsG1–G0vsG3	0.02	25,713	4.04	< 0.001
	G0vsG2–G0vsG3	0.03	25,713	5.67	< 0.001

**Table 2:** Summary of follow-up tests statistics for cosine similarity (CS) mean differences between emotions and within generation contrasts from SET2.

Emotion	Contrasts	Estimate	Degrees of Freedom	t-ratio	*p* value
SET2: G0vsG1, G1vsG2, G2vsG3					
Disgust	G0vsG1–G1vsG2	− 0.02	25,713	− 4.11	< 0.001
	G0vsG1–G2vsG3	− 0.04	25,713	− 8.97	< 0.001
	G1vsG2–G2vsG3	− 0.02	25,713	− 4.86	< 0.001
Embarrassment	G0vsG1–G1vsG2	− 0.01	25,713	− 2.02	0.04
	G0vsG1–G2vsG3	− 0.02	25,713	− 5.41	< 0.001
	G1vsG2–G2vsG3	− 0.02	25,713	− 3.39	< 0.01
Joy	G0vsG1–G1vsG2	− 0.01	25,713	− 2.04	0.04
	G0vsG1–G2vsG3	− 0.03	25,713	− 6.32	< 0.001
	G1vsG2–G2vsG3	− 0.02	25,713	− 4.28	< 0.001
Risk	G0vsG1–G1vsG2	− 0.05	25,713	− 11.28	< 0.001
	G0vsG1–G2vsG3	− 0.07	25,713	− 15.13	< 0.001
	G1vsG2–G2vsG3	− 0.02	25,713	− 3.85	< 0.001
Sadness	G0vsG1–G1vsG2	− 0.05	25,713	− 11.48	< 0.001
	G0vsG1–G2vsG3	− 0.06	25,713	− 14.09	< 0.001
	G1vsG2–G2vsG3	− 0.01	25,713	− 2.62	0.01

Inspecting the results in Table 1, stories of embarrassment and joy best maintained their emotional signature. Stories of sadness were also affectively well maintained, but began to deviate in generation three. There were exceptions for risk and disgust which had the largest generational shifts in similarities. Thus, risk and disgust appear to be least well preserved upon retellings. For comparison, recall that out-of-category comparisons among cosine similarities are all negative.

The follow up test results for SET2 in Table 2 indicate that stories from later generations become more and more similar to their immediate descendants. This suggests a convergence towards a definitive representation of the story emotion, as retellers refine the story for efficient transmission, which may be partly mediated by a decline in word count. Taking into account the results from SET1, it would appear that for the embarrassment emotion, not only was it well maintained by retellers, it was also refined into an efficient form for the next generation. This also holds true for the joy and sadness emotions. However, this may not be the case for risk and disgust, as the results from SET1 show that their retellings diverge more from the original.

## Discussion

We establish that RoBERTa is able to encode emotionally rich story embeddings. Given its emotion detection performance, and the analysis of its principal components, RoBERTa demonstrated that it is capable of modelling similar patterns to human evaluators offering emotional appraisals of story situations. RoBERTa could also identify intensities without explicit instruction, overcoming the orthogonality of the one-hot encoded classes.

RoBERTa’s performance was achieved even though our corpus included no emotion words specific to the category of membership. There were no words describing emotions explicitly dedicated to category membership in the seed stories. For example, the word *happy* occurs in stories communicating risk, rage, joy, and disgust. Nonetheless, later retellers could and did add emotion words. While RoBERTa may have connected some explicit words with specific emotions (for example, one of our sad seed stories contained a swimming competition and thus the word swimming mostly appeared in sad stories), these words alone do not explain the varying degrees of intensity of that emotion. For RoBERTa to encode emotions and intensity requires observing numerous variations of sequences and word combinations.

This study also sought to bring light to the question of whether individuals preserve the emotion of a story as they retell it to others. Our results indicate that people preserve emotions within their retellings, but this differs quantitatively for different emotions. Retellings maintained high degrees of similarity. This was true even though stories shrank substantially in total word count across retellings from an average of 182.7 words in the seeds to 50.2 words in the third generation, and thus suffered significant information loss. Embarrassment, joy, and sadness were most well preserved. Prior research on memory for emotions demonstrates that valence (positive and negative) and arousal (intensity), tend to be the most well remembered^46,47^. The present work is the first to demonstrate that this may also be preserved in story retellings.

We also found that stories were sequentially refined along the path of transmission. This is consistent with prior work showing that information passed through retellings becomes increasingly memorable, more so than the original information^48^. Iterated-learning through sequential learners has also been shown to reduce variability in the original information^49,50^, which is likely to facilitate accurate communication of the information to future learners.

Notably, preservation was least observed for risk and disgust. In addition to the leading role of valence (i.e., joy and sadness) described in prior work above, there are several possible explanations for why this might be the case.

One explanation revolves around people’s heterogenous perceptions towards those emotions. What one considers as disgusting or risky may not necessarily be felt in the same fashion by others. Indeed, word norms, in which individuals rate words along emotional dimensions show that disgust is less reliably rated across individuals compared with other emotions^51^. To our knowledge, there are no existing word norms for risk.

Another possibility is to consider the specific narrative structure of risk and disgust. It is possible that risk and disgust did not operate as thematic emotion episodes in the sense of Frijda et al.^25^. In the case of risk, the riskiness of the situation and the feelings of the risk-taking character did not match, as we noted above. It is thus possible that retellers collapsed this gap between situation and feeling by the character, thereby producing unclear signals. It is thus possible that RoBERTa picked up correct assessments since the retellings did not preserve riskiness well, a hypothesis also supported by the human raters in Breithaupt et al.^19^. We should note that risk is well preserved and in fact increased when retellers are prompted to pay attention to risk (see Moussaïd et al.^52^). Risk communication is important for alerting others^48^, but the corpus did not highlight this dimension. In the corpus, retellers may have focussed on other story elements. This idea also allows for the possibility to describe successful cases of emotion preservation as emotions of resolution (see Breithaupt et al.^34^).

Likewise, disgust rarely ends a story. In the context of our corpus, disgust occurred at the end of the story, as in cases with dirty roommates or exotic food, but while disgust may have signalled a warning to readers (see Strohminger^53^), it was not a resolution. Disgust could provide an ending if, for example, disgust would be connected to a punishment, but this was not the case in most of the stories in the corpus. Disgust may also have been poorly preserved since retellers may not have wanted to offend their audience or did not see a need for transmission of the disgusting story elements^54^.

Another possible explanation for the specific differences in emotion preservation we record could be that some emotions are connected with a specific function to reward or punish characters in a story or people in everyday communication. William Flesch^55^ proposed that the “comeuppance” function of fiction is a core function of narrative in general. Indeed, happy endings in the stories of the corpus reward people, not necessarily for morally positive behaviour, but also for mere endurance (the freshman in college is sticking it out, despite bad experiences). Sad stories and embarrassing stories often could make sense as punishment for specific behaviour (do not go to a swimming competition while your mother is severely ill). Embarrassment in particular is connected with shame, stigma, and social ostracising^54^. We list this theory as a potential explanation, though there are many stories that do not seem to fit the pattern, such as sad stories about a sick dog.

Our results are not meant to settle this question but to support more research in the important connection between narratives and emotion. More emotions need to be included in further studies, before a theoretical model can be substantiated. A limitation of our study is that it includes only one positive emotion (for classifications, see Graham et al.^56^; Keltner and Cowen^57^).

In summary, this study provides support for four conclusions: (1) RoBERTa can identify emotions in stories it was not trained on; (2) people preserve emotions during the process of story retelling, (3) the level of preservation differs by emotion type, and (4) preservation is strengthened through additional retellings.

The methods we use and the results are meaningful since they open the door for NLP research to focus on emotions in context-driven narratives without explicitly designating emotions. Emotions in everyday communication, social media, news reporting, and in literary fiction often do not emerge from explicit emotion words or short phrases, but rather require complex constructions of contexts. This work is a first step to tackle situation-driven emotions with machines. The findings that some, but not all emotions are preserved in story retelling is relevant since it allows prediction of which kind of information is more likely to be passed on, preserved, increased, or omitted.

The better understanding of the transmission of information is highly relevant for many domains, including education and learning, fact preservation, fake news communication and propaganda, social memory, moral or religious teaching, and information proliferation and evolution more generally (e.g., Hills^58^; Varnum and Grossmann^1^). Our research does not show that story preservation is better when it includes certain emotions. However, it does suggest that story information tied to some emotions leads to better preservation than story information tied to other emotions, which provides an important extension to prior work showing the influence of valence and arousal alone.

## Supplementary Information

Supplementary Figure 1.

## Data Availability

The datasets generated and/or analysed during the current study are available in the github repository, https://github.com/hejackhe/BDS-Project.

## References

[1] Varnum ME, Grossmann I (2017). Cultural change: The how and the why. Perspect. Psychol. Sci..

[2] Dunbar RIM (1998). Grooming, Gossip, and the Evolution of Language.

[3] Mesoudi A, Whiten A, Dunbar R (2006). A bias for social information in human cultural transmission. Br. J. Psychol..

[4] Damasio AR (2006). Descartes' Error.

[5] Kramer AD, Guillory JE, Hancock JT (2014). Experimental evidence of massive-scale emotional contagion through social networks. Proc. Natl. Acad. Sci..

[6] Bietti LM, Tilston O, Bangerter A (2019). Storytelling as adaptive collective sensemaking. Top. Cogn. Sci..

[7] Moore R, Hills TT (2022). The evolution of imagination and the adaptive value of imaginary worlds. Behav. Brain Sci..

[8] Eriksson K, Coultas JC (2014). Corpses, maggots, poodles and rats: Emotional selection operating in three phases of cultural transmission of urban legends. J. Cogn. Cult..

[9] Breithaupt F (2022). Das Narrative Gehirn. Was Neuronen erzählen.

[10] Rao N, Chu SL, Faris RW, Ospina D, Cardona-Rivera R, Sullivan A, Young R (2019). The effects of interactive emotional priming on storytelling: An exploratory study. Interactive Storytelling. ICIDS 2019. Lecture Notes in Computer Science.

[11] Elkins K (2022). The Shapes of Stories: Sentiment Analysis for Narrative.

[12] Reagan AJ, Mitchell L, Kiley D, Danforth CM, Dodds PS (2016). The emotional arcs of stories are dominated by six basic shapes. EPJ Data Sci..

[13] Nabi RL, Green MC (2015). The role of a narrative's emotional flow in promoting persuasive outcomes. Media Psychol..

[14] Bilandzic H, Kinnebrock S, Klingler M (2020). The emotional effects of science narratives: A theoretical framework. Media Commun..

[15] Dunlop S, Wakefield M, Kashima Y (2008). Can you feel it? Negative emotion, risk, and narrative in health communication. Media Psychol..

[16] Rimé B (2020). Emotions at the service of cultural construction. Emot. Rev..

[17] Rimé B (2009). Emotion elicits the social sharing of emotion: Theory and empirical review. Emot. Rev..

[18] Stubbersfield JM, Tehrani JJ, Flynn EG (2015). Serial killers, spiders and cybersex: Social and survival information bias in the transmission of urban legends. Br. J. Psychol..

[19] Breithaupt F, Li B, Liddell TM, Schille-Hudson EB, Whaley S (2018). Fact vs. affect in the telephone game: All levels of surprise are retold with high accuracy, even independently of facts. Front. Psychol..

[20] Loewenstein GF, Weber EU, Hsee CK, Welch N (2001). Risk as feelings. Psychol. Bull..

[21] Mandler JM, Johnson NS (1977). Remembrance of things parsed: Story structure and recall. Cogn. Psychol..

[22] Trabasso T, Stein NL, van den Broek PW, Bauer PJ, Bourg T (1997). Narrating, representing, and remembering event sequences. Developmental spans in event comprehension and representation: Bridging fictional and actual events.

[23] Zwaan RA, Langston MC, Graesser AC (1995). The construction of situation models in narrative comprehension: An event-indexing model. Psychol. Sci..

[24] Zacks JM (2020). Event perception and memory. Annu. Rev. Psychol..

[25] Frijda NH, Mesquita B, Sonnemans J, Van Goozen S (1991). The duration of affective phenomena or emotions, sentiments and passions. Int. Rev. Stud. Emot..

[26] Mikolov, T., Sutskever, I., Chen, K., Corrado, G. S. & Dean, J. Distributed representations of words and phrases and their compositionality. In *Advances in Neural Information Processing Systems*, 3111–3119 (2013).

[27] Mikolov, T., Chen, K., Corrado, G. & Dean, J. Efficient estimation of word representations in vector space. In *International Conference on Learning Representations*. arXiv preprint https://arxiv.org/abs/1301.3781 (2013).

[28] Le, Q. & Mikolov, T. Distributed representations of sentences and documents. In *Proceedings of the**International Conference on Machine Learning*, 188–1196 (2014).

[29] Hollis G, Westbury C (2016). The principles of meaning: Extracting semantic dimensions from co-occurrence models of semantics. Psychon. Bull. Rev..

[30] Vaswani, A., Shazeer, N., Parmar, N., Uszkoreit, J., Jones, L., Gomez, A. N., Kaiser, L. & Polosukhin, I. Attention is all you need. In *Advances in Neural Information Processing Systems*, 5998–6008 (2017).

[31] Devlin, J., Chang, M. W., Lee, K. & Toutanova, K. Bert: Pre-training of deep bidirectional transformers for language understanding. In *Proceedings of the 2019 Conference of the North American Chapter of the Association for Computational Linguistics: Human Language Technologies (NAACL/HLT)*, 4171–4186 (2018).

[32] Huang, Y. H., Lee, S. R., Ma, M. Y., Chen, Y. H., Yu, Y. W. & Chen, Y. S. EmotionX-IDEA: Emotion BERT—an Affectional Model for Conversation. *Proceedings of the 7th International Workshop on Natural Language Processing for Social Media*. Retrieved from arXiv preprint https://arxiv.org/abs/1908.06264 (2019).

[33] Acheampong FA, Wenyu C, Nunoo-Mensah H (2020). Text-based emotion detection: Advances, challenges, and opportunities. Eng. Rep..

[34] Breithaupt F, Li B, Kruschke JK (2022). Serial reproduction of narratives preserves emotional appraisals. Cogn. Emot..

[35] Bartlett FC (1932). Remembering: An Experimental and Social Study.

[36] Kashima Y (2000). Maintaining cultural stereotypes in the serial reproduction of narratives. Personal. Soc. Psychol. Bull..

[37] Mesoudi A, Whiten A (2008). The multiple roles of cultural transmission experiments in understanding human cultural evolution. Philos. Trans. R. Soc. Lond..

[38] Liu, Y., Ott, M., Goyal, N., Du, J., Joshi, M., Chen, D., Levy, O., Lewis, M., Zettlemoyr, L. & Stoyanov, V. RoBERTa: A robustly optimized BERT pretraining approach. arXiv preprint https://arxiv.org/abs/1907.11692 (2019).

[39] Huggingface. RoBERTa. *Transformers.* Retrieved from https://huggingface.co/transformers/model_doc/roberta.html (2020).

[40] Kingma, D. P. & Ba, J. ADAM: A method for stochastic optimization. In *Proceedings of the**International Conference on Machine Learning.* Retrieved from arXiv preprint https://arxiv.org/abs/1412.6980). (2015).

[41] Landauer T, Laham D, Foltz P, Jordan MI, Kearns MJ, Solla SA (1988). Learning human-like knowledge by singular value decomposition: A progress report. Advances in Neural Information Processing Systems 10.

[42] Cotterell, R., Poliak, A., Van Durme, B. & Eisner, J. Explaining and generalizing skip-gram through exponential family principal component analysis. In *Proceedings of the 15th Conference of the European Chapter of the Association for Computational Linguistics*. (Valencia, 2017).

[43] Singhal A (2001). Modern information retrieval: A brief overview. Bull. IEEE Comput. Soc. Tech. Comm. Data Eng..

[44] Huang, A. Similarity measures for text document clustering. In *Proceedings of the Sixth New Zealand Computer Science Research Student Conference (NZCSRSC2008*), 9–56 (Christchurch, 2008).

[45] Powers DMW (2011). Evaluation: From precision, recall and F-measure to ROC, informedness, markedness and correlation. J. Machine Learn. Technol..

[46] Adelman JS, Estes Z (2013). Emotion and memory: A recognition advantage for positive and negative words independent of arousal. Cognition.

[47] Buchanan TW (2007). Retrieval of emotional memories. Psychol. Bull..

[48] Jagiello RD, Hills TT (2018). Bad news has wings: Dread risk mediates social amplification in risk communication. Risk Anal..

[49] Reali F, Griffiths TL (2009). The evolution of frequency distributions: Relating regularization to inductive biases through iterated learning. Cognition.

[50] Smith K, Wonnacott E (2010). Eliminating unpredictable variation through iterated learning. Cognition.

[51] Stadthagen-González H, Ferré P, Pérez-Sánchez MA, Imbault C, Hinojosa JA (2018). Norms for 10,491 Spanish words for five discrete emotions: Happiness, disgust, anger, fear, and sadness. Behav. Res. Methods.

[52] Moussaïd M, Brighton H, Gaissmaier W (2015). The amplification of risk in experimental diffusion chain. Proc. Natl. Acad. Sci..

[53] Strohminger N (2014). Disgust talked about. Philos. Compass.

[54] Smith RA (2007). Language of the lost: An explication of stigma communication. Commun. Theory.

[55] Flesch W (2007). Comeuppance: Costly Signaling, Altruistic Punishment, and Other Biological Components of Fiction.

[56] Graham LE, Thomson AL, Nakamura J, Brandt IA, Siegel JT (2019). Finding a family: A categorization of enjoyable emotions. J. Posit. Psychol..

[57] Keltner D, Cowen A (2021). A taxonomy of positive emotions. Curr. Opin. Behav. Sci..

[58] Hills TT (2019). The dark side of information proliferation. Perspect. Psychol. Sci..

