# Supplementary material for: Quantifying the retention of emotions across story retellings
Source: Sci Rep. 2023 Feb 11;13:2448. doi: 10.1038/s41598-023-29178-8 (PMC9922315; doi:10.1038/s41598-023-29178-8)
Supplement: Supplementary file 1 — Supplementary Figure 1. [file 41598_2023_29178_MOESM1_ESM.docx]

**Supplementary Material**


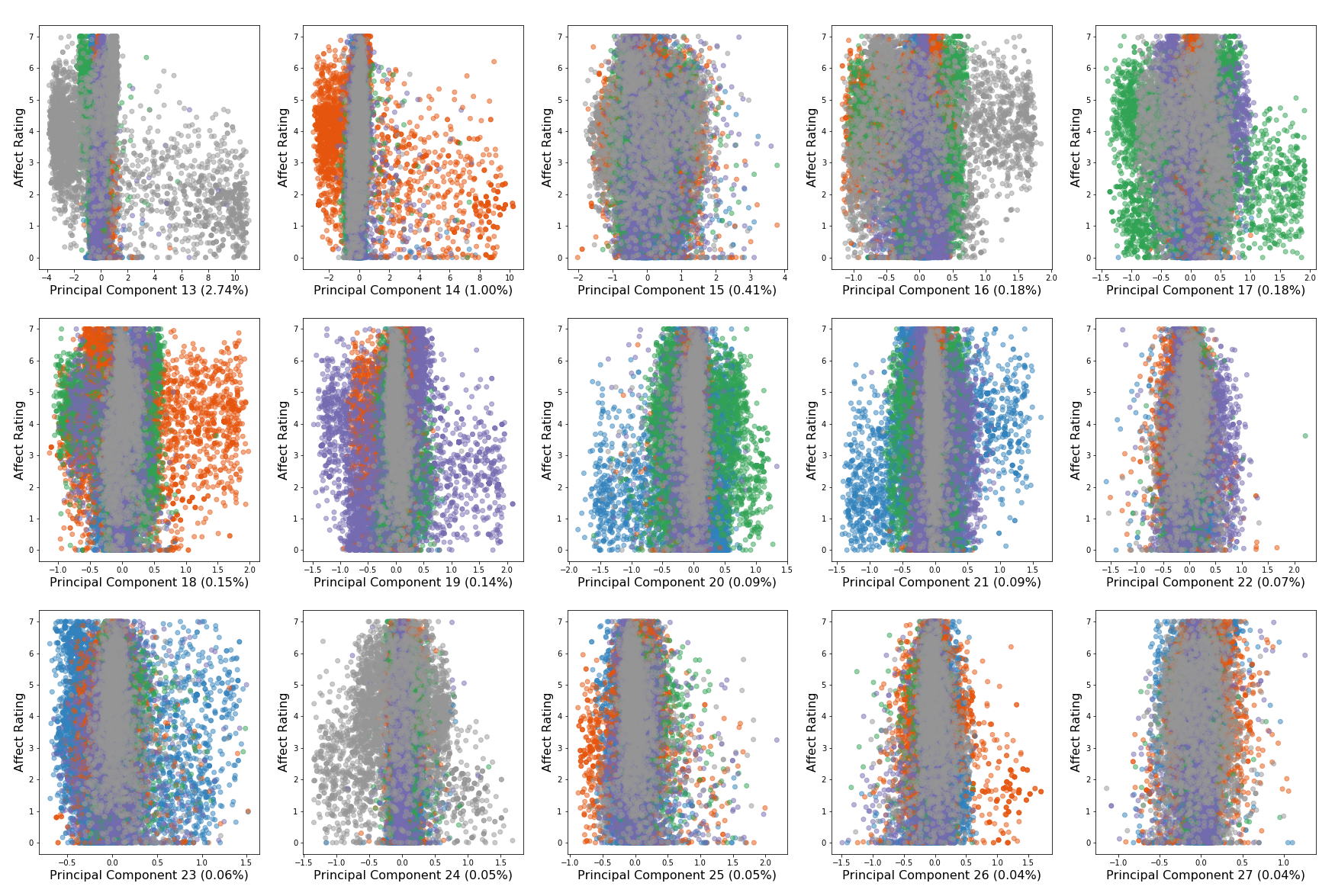


***Figure S1:*** Scatterplots for each of the remaining 27 PCs ranked by explained variance (in brackets), from all fine-tuned RoBERTa embeddings. Each subplot plots the projected PC scores on the x-axis against their corresponding affect ratings on the y-axis. Every observation is color-coded by emotion, with orange representing embarrassment stories, grey for sadness stories, green for joy, purple for risk and blue for disgust stories.
